# A Real-Space Study of Flat Bands in Nanowires

**DOI:** 10.3390/nano13212864

**Published:** 2023-10-29

**Authors:** Vicenta Sánchez, Chumin Wang

**Affiliations:** 1 Departamento de Física, Facultad de Ciencias, Universidad Nacional Autónoma de México, Mexico City 04510, Mexico; vicenta@unam.mx; 2 Instituto de Investigaciones en Materiales, Universidad Nacional Autónoma de México, Mexico City 04510, Mexico

**Keywords:** flat band, nanowire, convolution theorem, independent channels

## Abstract

The flat electronic band has remarkable relevance in the strongly correlated phenomena mainly due to its reduced kinetic energy in comparison to the many-body potential energy. The formation of such bands in cubically structured nanowires is addressed in this article by means of a new independent channel method and a generalized convolution theorem developed for the Green’s function including the first, second, and third neighbor interactions. A real-space renormalization method is further applied to address macroscopic-length aperiodic nanowires. We also determined the appearance condition of these flat bands, as well as their degeneracy and robustness in the face of perturbations, such as structural dislocations. Finally, the possible experimental detection of this flat band via the electronic specific heat is analyzed.

## 1. Introduction

In a crystalline solid, the null group velocity and infinite effective mass constitute the principal characteristics of a flat band, which is a highly degenerate energy level in the quantum mechanical point of view, in contrast to the massless Bloch electrons in two-dimensional hexagonal lattices. Heavy fermion metals represent a recognized example of flat bands formed by localized atomic orbitals [1]. Recently, topological flat bands originating from the geometrical interference of wave functions have significantly expanded the list of flat-band systems. The experimental realization of nearly flat bands has been achieved in Kagome materials, such as CoSn [2,3], Co_3_Sn_2_S_2_ [4], and YCr_6_Ge_6_ [5], as well as Moiré superlattices, such as twisted bilayer transition-metal dichalcogenides [6] and the twisted bilayer graphene [7], whose unconventional superconductivity observed at the magic angle has lately attracted significant attention [8]. In general, a large electronic density of states at the Fermi level induces a high superconducting transition temperature [9].

In recent decades, the research of materials at nanoscale has experienced exponential growth, revealing quantum phenomena in the macroscopic world. With a two-dimensional quantum confinement, the nanowires exhibit an exceptional itinerant ferromagnetism [10,11] and perform a crucial building-block role in nanoelectronics [12]. In this article, we report a real-space tight-binding study on the electronic band structure of nanowires built by cubically arranged atoms with interactions up to third neighbors. This study was carried out by means of a new independent channel method and a renewed convolution theorem, extending the previous one only for lattices with nearest neighbor interactions [13]. A real-space renormalization method [14] is further applied to address macroscopic-length aperiodic nanowires. The results show the formation of multiple flat electronic bands with a macroscopic number of degeneracies in each of them. These flat bands are robust under structural disorder perturbations, such as aperiodic dislocations, and can be observed by means of electronic specific heat measurements. 

## 2. The Model 

**Figure 1:**
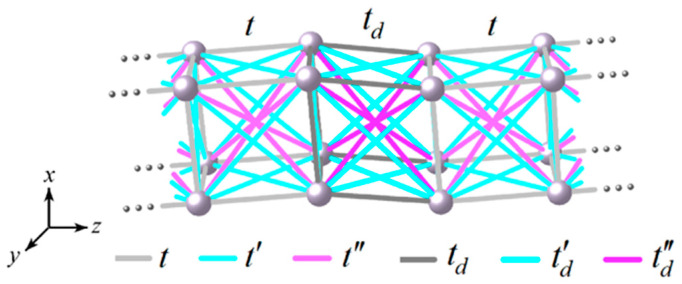
Schematic representation of a $2\times 2\times N_{z}$ nanowire with first (*t*), second $\left(t^{\prime }\right)$, and third $\left(t^{\prime \prime }\right)$ neighbor hopping integrals, as well as a dislocation characterized by $t_{d},\;{t^{\prime }}_{d}$, and ${t^{\prime \prime }}_{d}$.

Let us consider a cubically structured nanowire with electron hopping between first, second, and third neighboring atoms, as shown in Figure 1 for a nanowire of $N_{x}\times N_{y}\times N_{z}$ atoms with $N_{x}\;=\;N_{y}\;=2$ and a transversal dislocation defect at the center of the figure characterized by first ($t_{d}$), second (${t^{\prime }}_{d}$), and third (${t^{\prime \prime }}_{d}$) neighbor hopping integrals. The presence of such defects is common in nanowires since it very slightly alters the total free energy [15].

The band structure of spinless electrons in this nanowire can be studied by means of a tight-binding Hamiltonian with null on-site energies given by

(1) $$\hat{H}={\hat{H}}_{1}+{\hat{H}}_{2}+{\hat{H}}_{3}$$

where considering possible transversal dislocations along the Z direction we have

(2) $${\hat{H}}_{1}=\sum_{l,j,k}\left\{t|l,j,k\rangle \langle l\pm 1,j,k|+t|l,j,k\rangle \langle l,j\pm 1,k|+t_{k}|l,j,k\rangle \langle l,j,k+1|+t_{k-1}|l,j,k\rangle \langle l,j,k-1|\right\}$$

(3) $$\begin{array}{c} {\hat{H}}_{2}=\sum_{l,j,k}\left\{t^{\prime }|l,j,k\rangle \langle l\pm 1,j\pm 1,k|+t^{\prime }|l,j,k\rangle \langle l\pm 1,j\mp 1,k|+{t^{\prime }}_{k}|l,j,k\rangle \langle l\pm 1,j,k+1|\right.\\ \left.\;\;+{t^{\prime }}_{k-1}|l,j,k\rangle \langle l\pm 1,j,k-1|+{t^{\prime }}_{k}|l,j,k\rangle \langle l,j\pm 1,k+1|+{t^{\prime }}_{k-1}|l,j,k\rangle \langle l,j\pm 1,k-1|\right\} \end{array}$$

and

(4) $$\begin{array}{c} {\hat{H}}_{3}=\sum_{l,j,k}\left\{{t^{\prime \prime }}_{k}|l,j,k\rangle \langle l\pm 1,j\pm 1,k+1|+{t^{\prime \prime }}_{k}|l,j,k\rangle \langle l\pm 1,j\mp 1,k+1|\right.\\ \left.\;\;+{t^{\prime \prime }}_{k-1}|l,j,k\rangle \langle l\pm 1,j\pm 1,k-1|+{t^{\prime \prime }}_{k-1}|l,j,k\rangle \langle l\pm 1,j\mp 1,k-1|\right\} \end{array}$$

which respectively describe the electron hopping between first, second, and third neighboring atoms. In Hamiltonians (2), (3), and (4), *l*, *j*, and *k* are integer numbers correspondingly counting atoms along the X, Y, and Z directions with wavefunctions in the Dirac notation as $|l,j,k\rangle =|l\rangle |j\rangle |k\rangle$ [16], where $|l\rangle$, $|j\rangle$, and $|k\rangle$ are the one-dimensional Wannier functions [17].

Hamiltonian (1) with hopping integrals up to third neighbors can be rewritten as [18,19]

(5) $$\hat{H}={\hat{H}}_{x}⊗{\hat{I}}_{y}⊗{\hat{I}}_{z}+{\hat{I}}_{x}⊗{\hat{H}}_{y}⊗{\hat{I}}_{z}+{\hat{I}}_{x}⊗{\hat{I}}_{y}⊗{\hat{H}}_{z}+{\hat{H}}_{x}⊗{\hat{h}}_{y}⊗{\hat{I}}_{z}+{\hat{h}}_{x}⊗{\hat{I}}_{y}⊗{\hat{H}}_{z}+{\hat{I}}_{x}⊗{\hat{h}}_{y}⊗{\hat{H}}_{z}+{\hat{h}}_{x}⊗{\hat{h}}_{y}⊗{\hat{H}}_{z}$$

where symbol ⊗ denotes the Kronecker product, ${\hat{H}}_{p}\;=t\sum_{l}|l\rangle \;\langle l\;\pm \;1|$ and ${\hat{h}}_{p}\;=\tau \sum_{l}|l\rangle \;\langle l\;\pm \;1|$ with p=x or y, ${\hat{I}}_{q}=\;\sum_{l}|l\rangle \;\langle l\;|$ for q=x, y or z, and ${\hat{H}}_{z}\;=\;\sum_{k}\left(t_{k}|k\rangle \;\langle k\;+\;1|+t_{k-1}|k\rangle \;\langle k\;-\;1|\right)\;$, being τ a dimensionless hopping integral with $t^{\prime }\;=t\;\tau$, $t^{\prime \prime }\;=t\;\tau^{2}$, ${t^{\prime }}_{k}\;=t_{k}\tau$, and ${t^{\prime \prime }}_{k}\;=t_{k}\tau^{2}$. Now we project Hamiltonian (5) on the eigenstates $|\alpha ,\beta \rangle =|\alpha \rangle |\beta \rangle$ of the nanowire’s cross-section, which reduces it to an effective Z-directional Hamiltonian given by

(6) $${\hat{H}}_{z}^{eff}\;=\langle \alpha ,\beta |\hat{H}|\alpha ,\beta \rangle =E_{\alpha }{\hat{I}}_{z}\;+E_{\beta }{\hat{I}}_{z}\;+\;\;{\hat{H}}_{z}\;+E_{\alpha }e_{\beta }{\hat{I}}_{z}\;+e_{\alpha }\;{\hat{H}}_{z}\;+e_{\beta }\;{\hat{H}}_{z}\;+e_{\alpha }e_{\beta }{\hat{H}}_{z}$$

where $\alpha =1,\;2,\;\cdots ,N_{x}$, $\beta =1,\;2,\;\cdots ,N_{y}$, and [20]

(7) $$\left\{\begin{array}{c} E_{\alpha }=\langle \alpha |{\hat{H}}_{x}|\alpha \rangle =2t\cos[\alpha \pi /\left(N_{x}+1\right)]\\ E_{\beta }=\langle \beta |{\hat{H}}_{y}|\beta \rangle =2t\cos[\beta \pi /\left(N_{y}+1\right)]\\ e_{\alpha }=\langle \alpha |{\hat{h}}_{x}|\alpha \rangle =2\tau \cos[\alpha \pi /\left(N_{x}+1\right)]\\ e_{\beta }=\langle \beta |{\hat{h}}_{y}|\beta \rangle =2\tau \cos[\beta \pi /\left(N_{y}+1\right)] \end{array}\right.$$

Hamiltonian (6) can be rewritten as

(8) $${\hat{H}}_{z}^{eff}(\nu )=\varepsilon (\nu )\sum_{k}|k\rangle \langle k|+\sum_{k}\left[t_{k}(\nu )|k\rangle \langle k+1|+t_{k-1}(\nu )|k\rangle \langle k-1|\right]$$

where $\varepsilon (\nu )=E_{\alpha }\;+E_{\beta }\;+\;\;E_{\alpha }e_{\beta }=E_{\alpha }\;+E_{\beta }\;+\;\;e_{\alpha }E_{\beta }$ is the on-site energy and $t_{k}\;(\nu )=(1+e_{\alpha }\;+e_{\beta }\;+e_{\alpha }e_{\beta })\;t_{k}$ is the hopping integral of the ν-th Z-direction effective chain or channel with $\nu =1,\;2,\;\cdots ,N_{⊥}$ and $N_{⊥}\;=N_{x}\;\times N_{y}$. The condition for the existence of a fully disconnected channel with null hopping integrals or a highly degenerate flat band at energy equal to ε(ν) is

(9) $$1+e_{\alpha }\;+e_{\beta }\;+e_{\alpha }e_{\beta }\;=(1+e_{\alpha }\;)(1+e_{\beta }\;)=0$$

or

(10) $$e_{\alpha }\;=2\tau \cos\;[\alpha \pi /\left(\;N_{x}\;+1\right)]=-1\;\mathrm{or}\;e_{\beta }\;=2\tau \cos\;[\beta \pi /\left(\;N_{y}\;+1\right)]=-1$$

For example, a nanowire of $3\times 4\times N_{z}$ atoms with $N_{z}$ an arbitrary integer number has a flat band at $\varepsilon (\nu )=\;E_{\alpha }\;+E_{\beta }\;+\;\;e_{\alpha }E_{\beta }\;=\;E_{\alpha }\;=\;|\;t\;|\;\sqrt{2}$, if α=3 in (10), i.e., $t^{\prime }\;=\tau \;t=t\;/\sqrt{2}$ and $t^{\prime \prime }\;=\tau^{2}t=t\;/2$. Alternatively, if $t^{\prime }\;=t\;(\sqrt{5}-1)/2$ and $t^{\prime \prime }\;=t\;{\left(\sqrt{5}-1\right)}^{2}/4$ or β=4 in (10), the flat band will be located at $\varepsilon (\nu )=E_{\alpha }\;+E_{\beta }\;+\;\;E_{\alpha }e_{\beta }\;=E_{\beta }\;=\;|\;t\;|\;(\sqrt{5}+1)/2$. In the first case of $t^{\prime }\;=t\;/\sqrt{2}$, the degeneracy of the flat band is $4\times N_{z}$, while it is $3\times N_{z}$ when $t^{\prime }\;=t\;(\sqrt{5}-1)/2$, where $N_{z}$ is proportional to the length of nanowire. These degeneracy values were obtained from Equation (9). For instance, $\tau \;=1\;/\sqrt{2}$ is a solution of Equation (10) when α=3, and then there are four effective channels numbered by β=1, 2, 3 and 4 with all their hopping integrals equal to zero, which leads to a total degeneracy of $4\times N_{z}$.

On the other hand, the electronic density of states (DOS) can be calculated by means of the single-electron retarded Green’s function, $G(E)={\left(E-\hat{H}\right)}^{-1}$ determined by Hamiltonian (1) as [21]

(11) DOS(E)=−1πlimη→ 0+∑s=1NImGs,s (E+iη)

where $N=N_{x}\;\times N_{y}\;\times N_{z}$ is the total number of atoms in the nanowire and

(12) $$G_{\left(l,j,k),(l,j,k\right)}(\Theta )=\;\sum_{\alpha ,\beta ,\gamma }\frac{\langle l,\;j,k|\alpha ,\;\beta ,\gamma \rangle \langle \alpha ,\;\beta ,\gamma |l,\;j,k\rangle }{\Theta -E(\alpha ,\beta ,\gamma )}=\;\sum_{\alpha ,\beta ,\gamma }\frac{\langle l|\alpha \rangle \langle \alpha |l\rangle \langle j|\beta \rangle \langle \beta |j\rangle \langle k|\gamma \rangle \langle \gamma |k\rangle }{\Theta -E(\alpha ,\beta ,\gamma )}$$

with Θ=E+iη, $\hat{H}|\alpha ,\beta ,\gamma \rangle =E(\alpha ,\beta ,\gamma )|\alpha ,\beta ,\gamma \rangle$, $|\alpha ,\beta ,\gamma \rangle =|\alpha \rangle |\beta \rangle |\gamma \rangle$, ${\hat{H}}_{x}|\alpha \rangle =E_{\alpha }|\alpha \rangle$, ${\hat{H}}_{y}|\beta \rangle =E_{\beta }|\beta \rangle$ and ${\hat{H}}_{z}|\gamma \rangle =E_{\gamma }|\gamma \rangle$. Green’s function (12) can be rewritten as [13]

(13) G(l,j,k),(l,j,k)(Θ) = ∫− ∞∞dξ∫− ∞∞dζ∑γk|γγ|kΘ−E(ξ,ζ,γ)∑α,βl|αα|lj|ββ|jδ(ξ −Eα)δ(ζ −Eβ)      =1π2limη′,η″→ 0+∫− ∞∞dξ∫− ∞∞dζ∑γk|γγ|kΘ−E(ξ,ζ,γ)ImGl,l(ξ+iη′)ImGj,j (ζ+iη″),

where

(14) − limη′→ 0+ ImGl,l(ξ+iη′)/π=∑αl|αα|lδ(ξ−Eα)− limη″→ 0+ ImGj,j(ζ+iη″)/π=∑βj|ββ|jδ(ζ−Eβ)

Since $E(\alpha ,\beta ,\gamma )\;=\langle \alpha ,\beta ,\gamma |\hat{H}|\alpha ,\beta ,\gamma \rangle =\langle \gamma |{\hat{H}}_{z}^{eff}\;|\gamma \rangle \;=\;E_{\alpha }\;+\;E_{\beta }\;+\;\;E_{\alpha }e_{\beta }\;+(1+e_{\alpha }\;+e_{\beta }\;+e_{\alpha }e_{\beta })E_{\gamma }$, i.e., E(ξ,ζ,γ) is the eigenvalue of ${\hat{H}}_{z}^{eff}\;(\xi ,\zeta )$, Equation (13) becomes

(15) G(l,j,k),(l,j,k)(Θ)=1π2limη′,η″→ 0+∫− ∞∞dξ∫− ∞∞dζ Gk,keff(Θ,ξ,ζ)ImGl,l(ξ+iη′)ImGj,j (ζ+iη″)

Substituting (15) in (11), we obtain

(16) $$DOS(E)=\int_{-\;\infty }^{\infty }d\xi \int_{-\;\infty }^{\infty }d\zeta DOS_{z}^{eff}\;(E,\xi ,\zeta )\;DOS_{x}\;(\xi )\;DOS_{y}\;(\zeta )$$

or in the discrete form

(17) $$DOS(E)=\sum_{\nu =1}^{N_{⊥}}DOS_{z}^{eff}\;(E,\nu ),$$

where

(18) DOSzeff (E,ν)=−1πlimη→ 0+∑k=1NzImGk,keff (E+iη,ν)=−1πlimη→ 0+∑k=1NzImk1E−H^zeff (ν) +iηk

Equations (8), (17), and (18) will be used in the numerical calculations of DOS by means of a real-space renormalization procedure applied to each effective channel ν, whose recursive formulas are given in Appendix A of Ref. [13].

## 3. Results

**Figure 2:**
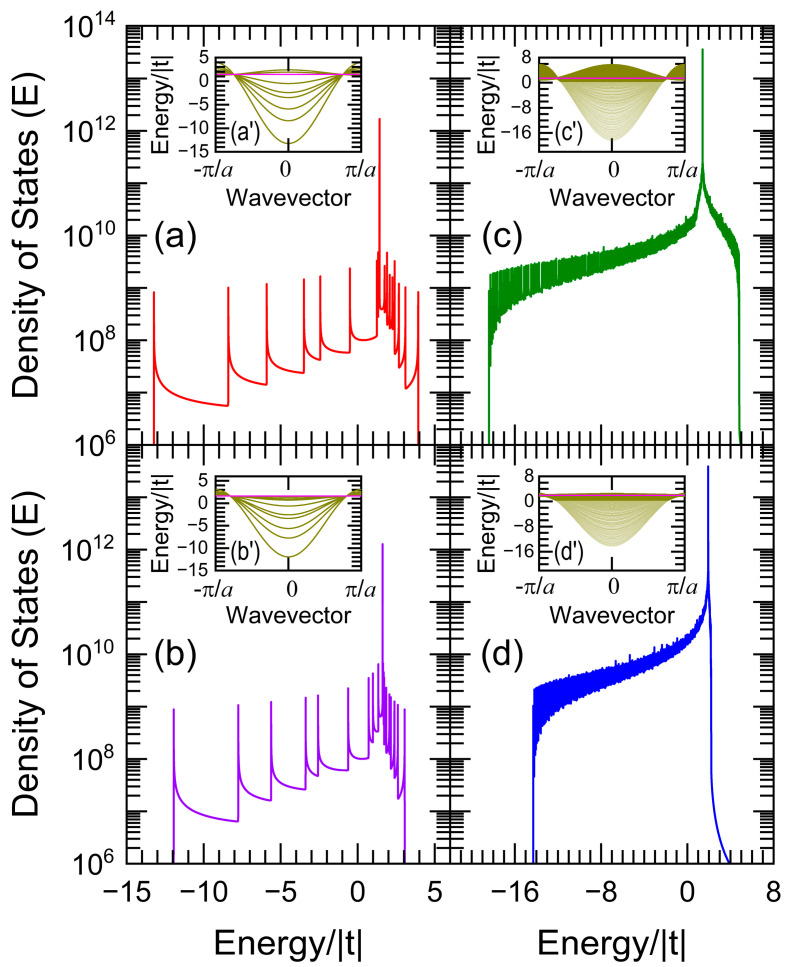
Density of states (DOS) versus energy (E) for periodic nanowires of (**a**,**b**) 3 × 4 × 134,217,728 and (**c**,**d**) 35 × 35 × 165,580,142 atoms with $\eta =10^{-4}\;|\;t\;|$, (**a**) $\tau =1\;/\sqrt{2}$, (**b**) $\tau =(\sqrt{5}-1)\;/\;2$, (**c**) $\tau =1\;/\sqrt{2}$, and (**d**) $\tau ={\left(2+\sqrt{3}\right)}^{-1/2}$. The corresponding dispersion relations or band structures are shown in the insets (**a′**–**d′**), whose flat bands are plotted using magenta color lines.

The electronic density of states (DOS) is plotted in Figure 2 as a function of the energy (E) with an imaginary part $\eta =10^{-4}\;|t|$ for periodic nanowires of $N_{x}\;\times N_{y}\;\times N_{z}$ atoms with (a, b) $N_{x}\;=3$, $N_{y}\;=4$, and $N_{z}\;=134217728$, while (c, d) $N_{x}\;=N_{y}\;=35$ and $N_{z}\;=165580142$, whose second and third neighbor hopping integrals are respectively $t^{\prime }\;=\tau \;t$ and $t^{\prime \prime }\;=\tau^{2}t$ with (a) $\tau \;=1\;/\sqrt{2}$, (b) $\tau \;=\left(\sqrt{5}-1\right)/2$, (c) $\tau \;=1\;/\sqrt{2}$, and (d) $\tau ={\left(2+\sqrt{3}\right)}^{-1/2}$.

The flat bands in Figure 2 are located at (a) $E\;=\sqrt{2}\;|\;t\;|$, (b) $E\;=\;|\;t\;|\;(\sqrt{5}+1)/2$, (c) $E\;=\sqrt{2}\;|\;t\;|$, and (d) $E={\left(2+\sqrt{3}\right)}^{1/2}\;|\;t\;|$, whose degeneracies are (a) 4 × 134,217,728, (b) 3 × 134,217,728, and (c,d) 35 × 16,558,042. These degrees of degeneracy were obtained from Equation (9). The rest of the peaks in Figure 2a–d are the van Hove singularities originating from each independent channel. The dispersion relations *E*(*k*), where *k* is the wavevector along the Z direction, are shown in the insets (a′–d′) using unit cells of (a′,b′) 3 × 4 and (c′,d′) 35 × 35 atoms, where the flat bands are plotted as magenta color lines.

The total electronic energy ($U_{el}$) for a given band filling (*ρ*) can be calculated as [22]

(19) $$U_{el}(T,\mu )=\int_{-\infty }^{\infty }E\;DOS(E)\;f_{FD}(E,T)\;\;dE$$

where $f_{FD}(E,T)={\left\{\;\exp[\left(E-\mu \right)/k_{B}T]+1\;\right\}}^{-1}$ is the Fermi–Dirac distribution with the Boltzmann constant $k_{B}$ and temperature *T*, whose chemical potential *µ* is determined by

(20) $$\rho =\frac{1}{N}\int_{-\infty }^{\infty }DOS(E)\;f_{FD}(E,T)\;\;dE.$$

In other words, for a given *ρ* and *T*, the chemical potential *μ* is firstly evaluated from (20) and then the total electronic energy $U_{el}$ is calculated using (19). The constant-volume electronic specific heat ($c_{el}$) per electron is given by

(21) $$c_{el}=\frac{1}{N_{el}}{\left(\frac{\partial U_{el}}{\partial T}\right)}_{V},$$

where

(22) $$N_{el}=\int_{-\infty }^{\infty }DOS(E)f_{FD}(E,T)\;dE$$

is the number of electrons in the nanowire for each band filling *ρ* that produces a specific chemical potential μ in the Fermi–Dirac distribution $f_{FD}(E,T)$.

**Figure 3:**
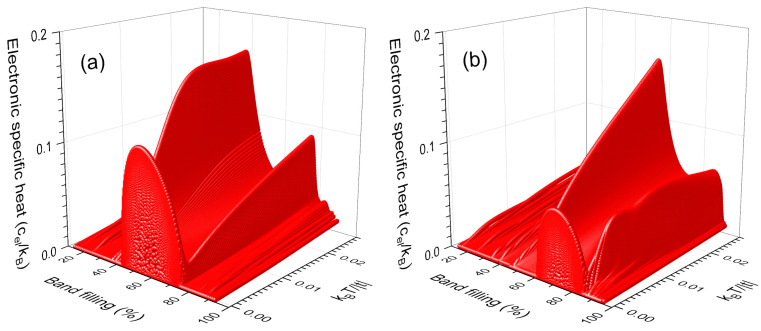
Electronic specific heat ($c_{el}$) versus the band filling (*ρ*) and temperature (*T*) for periodic nanowires of 3 × 4 × 134,217,728 atoms with (**a**) $t^{\prime }\;=t\;/\sqrt{2}$ and (**b**) $t^{\prime }\;=(\sqrt{5}-1)\;t/2$.

In Figure 3a,b, the electronic specific heat ($c_{el}$) as a function of the band filling (*ρ*) and temperature (*T*) is shown respectively for the nanowires of Figure 2a,b. Notice the maximums around T=0 come from the flat bands in DOS, whose presence can be analyzed using the Sommerfeld expansion for low temperatures given by [22]

(23) $$c_{el}(T)\approx \frac{\pi^{2}}{3}DOS(\mu )\;k_{B}^{2}\;T$$

i.e., the electronic specific heat linearly grows with *T*, whose slope is proportional to DOS(μ) and could be extremely large when *μ* is located at the flat band, as occurred at the maximums in Figure 3a,b. The difference of these two maximums in height and width is derived from that the flat band in Figure 3a contains a third part of the total electronic states, while in Figure 3b the flat band has only a fourth part. For example, the peak of $c_{el}(T\approx 0)$ in Figure 3a begins at band filling *ρ* ≃ 42.3% and ends at *ρ* ≃ 74.6%, while in Figure 3b it begins at *ρ* ≃ 60.5% and ends at *ρ* ≃ 84.7%. The ratio of these two peak widths is approximately 4/3. 

**Figure 4:**
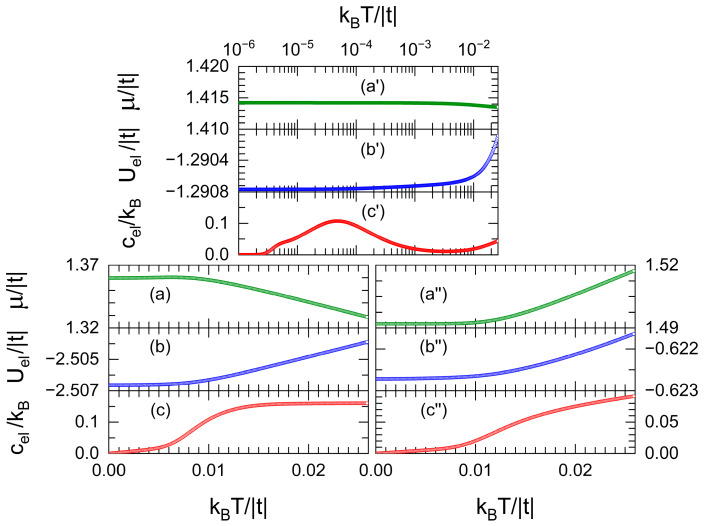
Chemical potential (*μ*), electronic energy ($U_{el}$) and specific heat ($c_{el}$) versus the temperature (*T*) for the same nanowire of Figure 3a with band fillings of (**a**–**c**) 40%, (**a′**–**c′**) 58%, and (**a″**–**c″**) 77%.

To illustrate more effects of the flat band, variations in the chemical potential (*μ*), electronic energy ($U_{el}$), and specific heat ($c_{el}$) as functions of the temperature (*T*) are plotted in Figure 4 for the same nanowire of Figure 3a with band fillings of (a–c) 40%, (a′–c′) 58%, and (a″–c″) 77%, respectively corresponding just below, at, and over the flat-band fillings.

In Figure 4b,b′,b″, the permanent growth of electronic energy $\left(U_{el}\right)$ with temperature (*T*) can be observed, since more electrons are excited into higher energy states for larger *T*, in contrast to the decreasing, almost constant, and increasing behaviors of the chemical potential (*μ*) respectively shown in Figure 4a,a′,a″. For example, the diminution of μ in Figure 4a is needed to keep a constant band filling of 40%, because such *μ* is located below the energy of flat-band states, and, at high temperature, a fraction of the total probability is shifted to larger energy states with a huge DOS, as stated by the Fermi–Dirac distribution. In a similar way, for *μ* larger than the flat-band energy, this shift of probability to higher energies with small DOS causes a growth of *μ* to preserve a constant band filling at large *T*.

Note in Figure 4c the peak of electronic specific heat ($c_{el}$) in logarithm scale draw when *μ* is located at the flat band. The rapid growth of this peak was discussed previously via Equation (23), while its quick decrease is related to the vanishing of occupation probability around *μ* at high temperatures predicted by the Fermi–Dirac distribution. In fact, the ubication of this peak close to $k_{B}T=10^{-4}\;|t|$ is associated with the imaginary part $\eta =10^{-4}\;|t|$ used in these calculations.

Now, let us introduce transversal dislocations with hopping integrals $t_{d},\;{t^{\prime }}_{d}\;\mathrm{and}\;\;{t^{\prime \prime }}_{d}$ into the nanowires, as shown in Figure 1. These dislocations are inserted following the Fibonacci quasiperiodic sequence $\left(F_{n}\right)$, i.e., $F_{n}=F_{n-1}⊕F_{n-2}$ with the initial conditions of $F_{1}=\;A$ and $F_{2}=\;A\;B$ [14], where ⊕ denotes the concatenation of two previous generations, *A* and *B* respectively represent hopping integrals *t* and $t_{d}$. In addition, we will keep the first *m* generations of the Fibonacci sequence without dislocations and introduce the first $t_{d}$ in the generation m+1, placing it at the union of generations m and m−1. For example, if m=2, we have $F_{1}=\;A$, $F_{2}=\;A\;A$, $F_{3}=\;A\;A\;B$, $F_{4}=\;A\;A\;BA\;A$, etc. 

**Figure 5:**
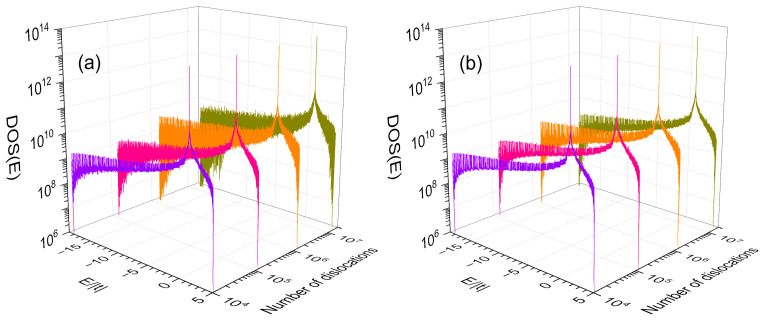
Electronic density of states (*DOS*) versus energy (*E*) with $\eta =10^{-4}\;|\;t\;|$ for nanowires of 35 × 35 × 165,580,142 atoms, 10,947 (violet line), 121,394 (pink line), 1,346,270 (orange line), and 14,930,353 (dark-yellow line) dislocations with $t^{\prime }\;=t\;/\sqrt{2}$, $t^{\prime \prime }\;=t\;/2$: (**a**) $t_{d}\;=0.5\;t$ and (**b**) $t_{d}\;=0.9\;t$.

In Figure 5, the electronic density of states (*DOS*) is plotted as a function the single-electron energy (*E*) with an imaginary part of $\eta =10^{-4}\;|\;t\;|$ for nanowires of 35 × 35 × 165,580,142 atoms containing 10,947 (violet line), 121,394 (pink line), 1,346,270 (orange line), and 14,930,353 (dark-yellow line) dislocations that respectively correspond to m=35,  30,  25 and 20. The second- and third-neighbor hopping integrals of these nanowires are respectively $t^{\prime }\;=t\;/\sqrt{2}$ and $t^{\prime \prime }\;=t\;/2$, while their dislocations are characterized by hopping integrals (a) $t_{d}\;=0.5\;t$, ${t^{\prime }}_{d}\;=0.5\;t/\sqrt{2}$, and ${t^{\prime \prime }}_{d}\;=0.5\;t/2$, as well as (b) $t_{d}=0.9\;t$, ${t^{\prime }}_{d}\;=0.9\;t/\sqrt{2}$, and ${t^{\prime \prime }}_{d}\;=0.9\;t/2$. The length of such nanowires corresponds to a Fibonacci sequence of generation n=40.

The high frequency oscillations of DOS along whole spectrum can be observed, whose amplitude grows with the number of dislocations, except the flat-band states remain intact. These oscillations originate from the presence of dislocations and are more notable when the difference $\left|t_{d}-t\right|$ grows.

## 4. Conclusions

This article presented a new independent channel method for cubically structured nanowires with first, second, and third neighbor hopping interactions, as well as a generalized convolution theorem for the single-electron Green’s function of such nanowires. The former analytically demonstrates the possible existence of flat bands and provides their appearance conditions, as well as their degrees of degeneracy, while the latter permits an efficient calculation of the density of states (*DOS*), as well as other physical quantities based on the Green’s function, such as the electrical conductivity via the Kubo–Greenwood formula [13]. 

As an example, a detailed study of a narrow nanowire of $3\times 4\times N_{z}$ atoms, where $N_{z}$ is a macroscopic integer number, was reported, whose *DOS* spectrum confirms the existence of a flat band with a macroscopic degree of degeneracy, and the presence of such a flat band may significantly alter the spectrum of electronic specific heat ($c_{el}$). This new spectrum of $c_{el}$ derived from the flat band can be experimentally measured, if we were able to synthesize nanowires that nearly satisfy the analytical relationship found in this article between the first, second, and third hopping-integral strengths by applying an external pressure, while the variation in band filling could be achieved via the application of a gate voltage. The assumption of $t^{\prime }/t=\tau$ and $t^{\prime \prime }/t=\tau^{2}$ can be closely satisfied, if the hopping integral scales almost exponentially with the interatomic distance (*d*) such as $t(d)\propto e^{-d/d_{0}}$, which leads to $t^{\prime }/t=e^{(\sqrt{2}-1)a/d_{0}}$ and $t^{\prime \prime }/t=e^{(\sqrt{3}-1)a/d_{0}}$ for s orbitals, where *a* is the nearest-neighbor interatomic distance and $d_{0}$ is a reference length. Hence, a hydrostatic external pressure may simultaneously modify the ratios $t^{\prime }/t$ and $t^{\prime \prime }/t$ to eventually satisfy the flat-band appearance condition such as $\tau =1/\sqrt{2}$ in the analyzed cases.

Finally, the results of this article also reveal the robustness of such a flat band in spite of the geometrical interference of wave functions induced by structural perturbations, such as the transversal dislocations that frequently appear in nanowires since they very slightly alter the total free energy. It would be worth mentioning that the independent channel method and convolution theorem reported in this article combined with the renormalization method [14] permit the development of a real-space quantum theory of solids and devices containing defects and structural interfaces, such as PNP and NPN transistors, as well as segmented nanowires for thermoelectric applications [23]. This development is currently in progress.

## Data Availability

Data sharing not applicable.
